# Abstract of Meteorological Observations for April, 1856, Made at Philadelphia, Pa.

**Published:** 1856-06

**Authors:** James A. Kirkpatrick


					﻿•g	Barometer. Thermom.i	'	----————
----------------------------1	fl
iqrfi	Mean	Mean! Rel. Force of Dew Rain &	Remarks.
2L	. 0	Daily Daily Daily Daily Humid. Vapor Point Melted Prevaih’ng	jq
C ~	-April. Mean Range. Mean Range 2 P.M. 2 P.M. 2P.M. Snow. Winds.	?->
£	______________________________________ ___________________________________ fl
O	Inches. Inches. Deg. Deg. Hunds. Inches. Deg. Inches. Poi.ts.	q
S 2s	M	9	;q24n	951	460	ta9	ao	'167	30 *1	1	WNW.	Clear. Barometer highest 30.273. Thermometer lowest 23°.	o
eot- O	3 29M6 *358 58 2 !92 to *3?I 50*8 } °-290	Cloudy; rain all day*
tn	Al	xht	* 1tU	48*2	inn	18	194	3 *~	vw’	Morning rain; afternoon and evening clear.	g
S	i	™	not	M7	10/2	v	310	N3’	Morning and afternoon cloudy; evening clear.
75	£	7	29.895 .147 52.7 5.8	51	.283 43.5	SW/ g “ar	fc
g	8	30.069 .174 57.0 4.3	43	.304 45.4	SW.	’
"-J .C	10	29 981 103 54 0 7*8	26	138 25*6	w * Morning cloudy; afternoon and evening dear.	2
D 11 29.S .104 50.3 3.?	3!	176 30	Mormng and afternoon clear; evening cloudy.	§
12	29,496 -484 66.2 15.8	52	.436 55.2	SW. Cloudy; morning and evening a few drops of rain. 10, P.M. to 10}, P.M. a §
„ =	„	90006 471 47 0 ’0 9	35	135 25 1	XTTrr wind hurricane from W. and N.W. with thunder and lightning.	pq
§§ 3	g	SS :S S -g	g	gg;	ja«
is 3 K SS :1’»S= « “ S °-883 (Vg'J	7
?	17	29.692	.184	53.7	3.0	71	-867	50.5	0.318	(Var.)	Rain all dav
Cl Ph	18	29.668	-196	67,9	3.3	43	-2o6	40.9	W.	Morning clear; afternoon and evening	cloudy.	3
CO O	19	29.748 .081 60./ 3.7	3o	-262 41.5	0.060 SE.	Cloudy; night	rain.	° J	g
O 20	29.607	.141	47.8	13.5	88	.292	44.3	0-063	(Var.)	Cloudy; rain all day.	S
P-<	21	29.380	.2l7	44.2	4.7	100	•299	4a.O	1‘232	(Var.)	Cloudy; rain all day.	Barometer	Zowest 29.277.	3
g >9	22	29.526 .242 46.0 3.2	78	.272 4.5	0.007 SW. Morning ram; afternoon cloudy; evening clear.	®
42 CQ	23	-9./03 .1<7 a3.7	7.7	76	-389 51.4	0.073	sw.	Cloudy; afternoon rain.	-fl
g5,®	of 299?2 203 602 n« 57	*416 539	0*037	CIoudy; 8i-P-M-th. and ltg.; 9, P.M., rain; th. and ltg. stopped at 11, P.M.	•
.	25	29.9J2 .203 60.2	0.8	57	1416 68,9	°-037	NE.	Morning and afternoon cloudy;	afternoon rain: evening clear.	°
£	C	26	fn n« ’if- 27	%	<Var')	Morning and evening clear; afternoon cloudy.	«*>
Cs . .3	([27	30.025 .13a 61.3	5.5	49	.370 50.7	SW.	Clear.	n
•S	2	28	29.785 -240	68.5 7.2	52	469 57.2	SW.	Morning and afternoon cloudy; evening clear.	5 ~
S	29	29.764 042 74.3	5.8	28	.28143.3	NW.	Morning and afternoon	cloudy; evening clear. Thermometer highest 80°.
30 29.930 .166 63.5 10.8	54	. 337 48.1 0.005 NE.	Cloudy; afternoon rain.	§
‘	Sa
o _----------------------------------------------------------------------------------------------------------------------------------------------| g
R Means) 1856 29,814 -18° 54,5 6,6 52 -29° 42,7 3’149 S.73° 37'W. 22-100	3 H
•h B P for > ------------------------------------------------------------------------- <U a>
J?April, ) 5 yrs. 29.809 .189 51.4 7.0 51 .271 40.9 4.752 N. 65° 55'W.18-100.	2° 43
~	»	t-t ■H>
---------------------------------------------------------------------------1----------------------------------------------------------__ a
				

